# Effects of Aerobic Exercise on Cytokine Expression in a Breast Cancer Mouse Model

**Published:** 2020-01

**Authors:** Bangsub LEE, Wooyoung CHUNG

**Affiliations:** Department of College of General Education, Chung-Ang University, Seoul, Republic of Korea

**Keywords:** Breast cancer, C-reactive protein, Cytokine, Inflammation, Interleukin

## Abstract

**Background:**

Regular physical activity lowers or prevents the risk of heart disease, diabetes, some cancers, the development of hypertension, and death from these diseases through a reduction in inflammation. Cytokines, such as interleukin (IL)-6, IL-18, tumor necrosis factor-alpha (TNF-*α*), and C-reactive protein (CRP) are major markers representing the inflammatory process. This study aimed to investigate cytokine mRNA expression levels of IL-6, IL-18, TNF-*α*, and CRP in hepatocytes from breast cancer xenograft mice with or without moderate exercise.

**Methods:**

Each of the 5 mice at SP Korea Company, Seoul, Korea in 2015 were randomly divided into 3 groups: control (CTL), breast cancer (BC), and breast cancer exercise (BCEX). The inflammatory markers were analyzed in 10-week-old female Balb/C nude mice hepatocytes (n = 15; CTL = 5, BC = 5, BCEX = 5). Moderate intensity physical activity in mice was performed on a treadmill at an intensity of 18 m/min for 12 weeks, at 30 min for 5 days per week.

**Results:**

IL-6, IL-18, TNF-*α*, and CRP mRNA expression levels of the BCEX group were significantly decreased compared to those of the BC group (*P* < 0.05), with no difference to the CTL group.

**Conclusion:**

There might be a reduced inflammatory process via a reduction in TNF-*α*, IL-6, IL-18, and CRP expression in breast cancer mice that were subjected to moderate intensity exercise.

## Introduction

Physical inactivity accounts for 4% of the world's deaths and is the fourth leading cause of death in the world (1). In particular, physical inactivity has become a risk factor for certain cancers. In fact, the American Cancer Society found that the incidence of cancer in adults under the age of 50 was due to a sedentary lifestyle, and a sedentary lifestyle resulted in a variety of physical disabilities (1).

Breast cancer is the second most common cancer in the United States and is the leading cause of cancer-related deaths. While breast cancer is a major cancer with underlying genetic causes, including hereditary breast cancer and ovarian cancer syndrome (HBOC) (2), most cases reported are associated with low physical activity and lifestyle risk factors. Recent animal studies have reported a relationship between physical activity and reduced breast cancer risk. Sports medicine and cancer-related studies have reported that physical activity in epidemiological studies and experiments is closely related to reduced risk of various cancers (1,3). However, studies on the prevention and treatment of cancer through physical activity have not yet been extensively performed.

Recent studies have reported that low-intensity exercise reduces abnormal cancer cell growth in colorectal cancer mouse models (4), and that regular exercise reduces tumor growth in breast and colorectal cancer mouse models. Zimmer et al emphasized the importance of exercise to increase the level of physical activity in breast cancer patients, suggesting that physical activity is associated with reducing fatigue and inflammation (5). The beneficial effects of physical activity on cancer development are known in many studies (6), and research on the mechanisms of cancer and physical activity is being actively studied (7). However, the mechanisms involved linking physical activity to cancer risk are complex and are likely to be due to a variety of causes, given the various effects of exercise.

Thus, due to various factors (obesity, fatty liver, energy balance, adipokines, insulin, estrogen, immune function, and inflammation), the mechanisms responsible for the beneficial effects of physical activity on cancer risk are likely complex and multifaceted (1). Recently, a reduction in inflammation caused by physical activity may play an important role (8). Inflammation is involved in most processes of cancer development and progression, and physical activity has been reported to reduce the inflammatory process (9).

However, it is still unclear whether the benefits of physical activity on inflammation in cancer patients directly affects the inflammatory pathways that are important for cancer growth, or whether it is simply a side effect of the established relationships between physical activity and cancer.

Therefore, we aimed to investigate the effects of exercise on inflammatory cytokines interleukin (IL)-6, IL-18, tumor necrosis factor-α (TNF-*α*), and C-reactive protein (CRP) in a breast cancer mouse model. The purpose of this study was to investigate the effects of 12 weeks of running on the cytokines IL-6, IL-18, CRP, and TNF-*α* in mice.

## Methods

### Subjects and experimental protocol

The ten-week-old Balb/c nude mice at birth were obtained from Charles River Corp (Tokyo, Japan) at the SP Korea Company, Seoul, Korea in 2015. Five mice were placed in individual cages and the contrast period was adjusted. The temperature and humidity were adjusted to 22 °C and 51%, respectively. Each of the 5 mice were randomly divided into 3 groups: normal mice (CTL), breast cancer (BC), and breast cancer exercise (BCEX). Diets were prepared from Purina Mills Inc. (MO, USA). The training group used mouse treadmills (MYUNGIN INSTRUMENTS CO., Seoul, Republic of Korea). The exercise protocol was as follows: mice ran for 30 min, at a speed of 18 m/min for 5 days per 12 weeks. The intensity of the exercise was set to 70%∼75% VO_2_ max.

All study procedure were approved by SP Korea Company ethically based on Helsinki Declaration.

### Cell culture

Cell lines were obtained from the American Type Culture Collection (ATCC, Manassas, VA). Cells were maintained in Dulbecco's modified Eagle's medium (Mediatech, Herndon, VA) containing 10% fetal bovine serum (FBS; HyClone, Logan, UT) and 1% antibiotics (GIBCO BRL, Carlsbad, CA).

### Tumor xenograft mice model

The MCF-7 human breast cancer cell line was used as a model for human breast cancer. Cells were maintained in a 37°C and 5% CO_2_ incubator. For inoculation, MCF-7 cells were resuspended in a mixture of Matrigel (Matrigel, BD Biosciences; Chicago, IL: Lot # 005002, 14.6 mg/mL) and Hank's Balanced Salt Solution (HBSS) to a final concentration of 25×10^6^ cells/mL. Cells were subcutaneously injected at a final dose of 5×10^6^ cells/0.2 mL/mouse.

For subcutaneous injection into the abdominal side of the abdomen, 23-gauge needles were used. The size of the tumor was measured after 14 days using an electronic caliper (Fowler Instruments; Newton, MA). Mice were randomly divided into two groups based on tumor size. There was no significant difference between mean starting tumor volumes for all groups within the study. Tumor measurements and observations were recorded 5 times a week. Tumor volume was calculated using the standard formula (length × width × width) × 0.5.

### Liver microsome preparation

The liver of breast cancer mice was weighed and homogenized in buffer (0.1 M potassium phosphate buffer containing 0.125 M potassium chloride, 1.0 mL EDTA, and protease inhibitor mixture (Sigma), pH 7.4). The mixture was homogenized and centrifuged at 13,000 × *g* for 20 min at 4 °C. The post-mitochondrial fraction was ultra-centrifuged at 250,000 × *g* for 45 min at 4 °C to collect microsomes. The pellet was resuspended in 10 mL Tris acetate buffer (pH 7.4), containing 0.1 ml EDTA and 23% glycerol. Samples were stored at −80 °C.

The protein concentration was measured with a bicin choninic acid (BCA) protein assay kit (Thermo Fisher Scientific Inc., Rockport, IL) with bovine serum albumin.

### Real-time PCR

The livers of the mice were weighed and RNA was isolated using RNA-Bee separation reagent (Tel-Test Inc., Friendswood, TX). A total of 1 μg RNA was used for cDNA synthesis using a high capacity cDNA storage kit (Applied Biosystems, Foster City, CA). Real-time PCR was performed with SYBR® Green PCR Master Mix (Applied Biosystems) using an Eppendorf Mastercycler ep. realplex PCR system. The normalization control was glyceraldehyde 3-phosphate dehydrogenase (GAPDH) mRNA.

### Primer sequences

MWG Biotech, Inc. (High Point, NC) custom synthesized PCR primers at a 50 nmol synthesis scale. The primers were desalted and lyophilized, and then diluted to 100 mL and stored at −80 °C (Table 1).

**Table 1:** Primer sets used for qPCR

***Genes***	***Forward***	***Reverse***
TNF-*α*	5′-GGCAGGTCTACTTTGGAGTCATTGC-3′	5′-ACATTCGAGGCTCCAGTGAATTCGG-3′
IL-6	5′-CCGGAGAGGAGACTTCACAG-3′	5′-GGAAATTGGGGTAGGAAGGA-3′
IL-18	5′-GCAGCAGGTGAGTGGGCAGT-3′	5′-CTGTACGCCTGGTTCGCTCTGT-3′
CRP	5′-AGCCTCTCTCATGCTTTTGG-3′	5′-TGTCTCTTGGTGGCATACGA-3′
GAPDH	5′-ACCACAGTCCATGCCATCAC-3′	5′-CACCACCCTGTTGCTGTAGCC-3′

### Statistical analysis

All data were analyzed as the mean ± standard error. All analyses were conducted using SPSS version 22.0 (IBM Corp., Armonk, NY, USA). A one–way analysis of variance and Kruskal-Wallis test, and a Tukey post-hoc test, were used to examine the differences in subject characteristics between the groups. The statistical significance level was set at *P* < 0.05.

## Results

### Inhibitory effect of exercise on TNF-α expression in the livers of breast cancer mice

**Fig. 1:**
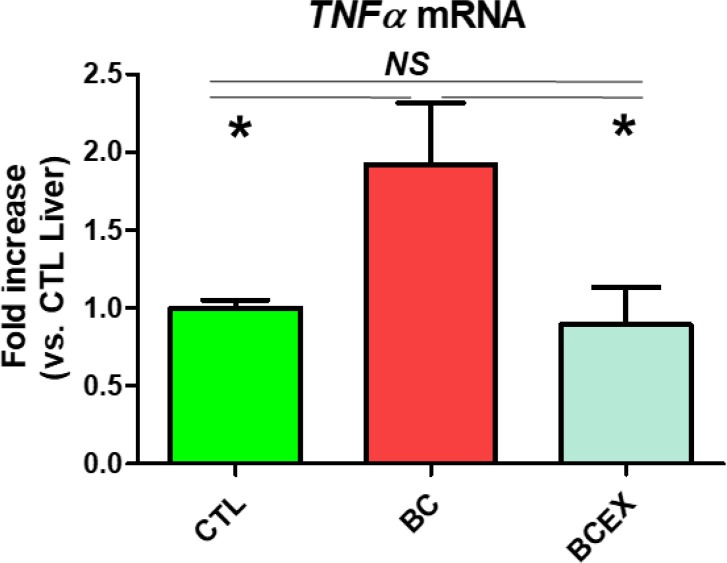
Expression of TNF-*α* in the liver. CTL, normal Balb/c nude mice group; BC, breast cancer mouse group; BCEX, breast cancer exercise group. Values are means ± standard error; n = 5 for each group; *P < 0.05, tested by one–way analysis of variance; NS, nonsignificant

TNF-*α* mRNA expression is presented in Fig. 1. TNF-*α* was significantly increased in the breast cancer group (BC: 1.91 ± 0.39) compared to the normal mouse group (CTL: 1.00 ± 0.05, *P* < 0.03). The breast cancer exercise group (BCEX: 0.89 ± 0.24) was significantly decreased compared to the breast cancer group (BC: *P* = 0.043) and showed no significant difference to the normal mouse group. The results show that long-term exercise reduces TNF-*α* mRNA expression in breast cancer mice, and long-term running can inhibit TNF-*α* in the liver.

### Inhibitory effect of exercise on IL-6 expression in the livers of breast cancer mice

**Fig. 2:**
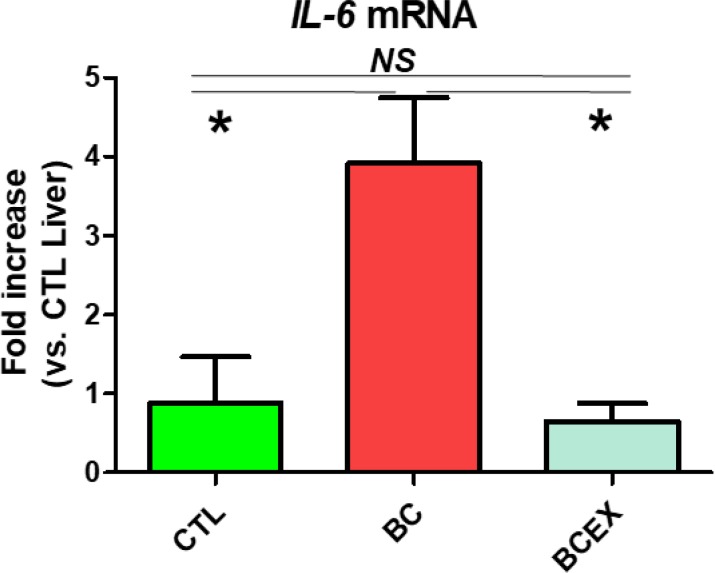
Expression of IL-6 in the liver. CTL, normal Balb/c nude mice group; BC, breast cancer mouse group; BCEX, breast cancer exercise group. Values are means ± standard error; n = 5 for each group; *P < 0.05, tested by one–way analysis of variance; NS, nonsignificant

IL-6 mRNA expression is presented in Fig. 2. IL-6 levels were significantly increased in the breast cancer group (BC: 3.91 ± 0.83) compared to the normal mouse group (CTL: 1.00 ± 0.63, *P* = 0.010). After 12 weeks of exercise, IL-6 mRNA expression in the breast cancer exercise group (BCEX: 0.90 ± 0.28) was significantly decreased compared to that of the breast cancer group (BC: *P* < 0.001), and there was no significant difference compared to the normal mouse group. The results show that long-term exercise reduces IL-6 mRNA expression in breast cancer mice, and long-term running can inhibit IL-6 in the liver.

### Inhibitory effect of exercise on IL-18 expression in the livers of breast cancer mice

**Fig. 3:**
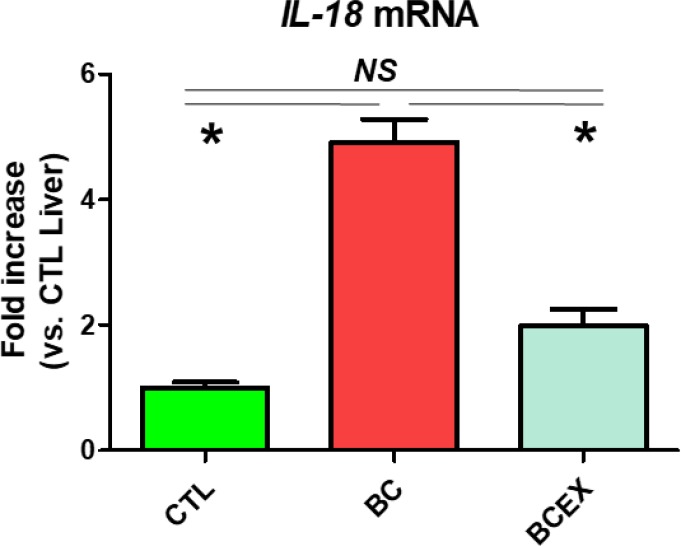
Expression of IL-18 in the liver. CTL, normal Balb/c nude mice group; BC, breast cancer mouse group; BCEX, breast cancer exercise group. Values are means ± standard error; n = 5 for each group; *P < 0.05, tested by one–way analysis of variance; NS, nonsignificant

IL-18 mRNA expression is presented in Fig. 3. IL-18 levels were significantly increased in the breast cancer group (BC: 4.91 ± 0.36) compared to the control group (CTL: 1.00 ± 0.09, *P* < 0.001). After 12 weeks of exercise, IL-18 mRNA expression was significantly decreased in the breast cancer exercise group (BCEX: 1.98 ± 0.27) compared to the breast cancer group (BC: *P* < 0.001), and there was no significant difference between the CTL group and BCEX group. The results show that long-term exercise reduces IL-18 mRNA expression in breast cancer mice, and long-term running can inhibit IL-18 in the liver.

### Inhibitory effect of exercise on CRP expression in the livers of breast cancer mice

**Fig. 4:**
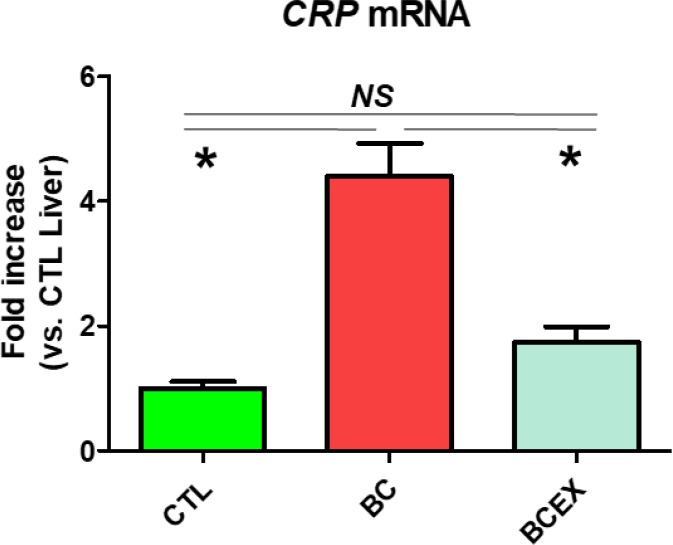
Expression of CRP in Liver. CTL, normal Balb/c nude mice group; BC, breast cancer mouse group; BCEX, breast cancer exercise group. Values are means ± standard error; n = 5 for each group; *P < 0.05, tested by one–way analysis of variance; NS, nonsignificant

C-reactive protein (CRP) mRNA expression is presented in Fig. 4. CRP levels were significantly increased in the breast cancer group (BC: 4.39 ± 0.53) compared to the normal control group (CTL: 1.00 ± 0.11, *P* < 0.001). CRP mRNA expression was significantly reduced in the 12-week exercise group with breast cancer (BCEX: 1.74 ± 0.25) compared to the breast cancer group (*P* < 0.001), and there was no significant difference to the CTL group. The results show that long-term exercise reduces CRP mRNA expression in breast cancer mice, and long-term running can inhibit CRP in the liver.

## Discussion

Medium intensity physical activity is known to be an effective means of maintaining health, and is effective in reducing the incidence of breast cancer and the effects of various factors on cancer (10,11). Insufficient physical activity may result in a higher breast cancer rate than that of women who have sufficient physical activity, and sedentary lifestyles may increase breast cancer risk (10,12). In this study, 12 weeks of running was performed in a mouse model of medium-intensity exercise; groups consisted of non-breast cancer bearing group without exercise (control group), BCEX, and BC. In order to investigate how exercise in breast cancer affects inflammatory cytokines, IL-6, IL-18, TNF-*α*, and CRP mRNA expression levels were analyzed in the livers of the study mice.

Inflammation has been reported to play an important role in the development and progression of cancer. Inflammatory substances that have the greatest effect on breast cancer include TNF-*α* and IL-6 (13,14). There are a variety of mechanisms that reduce the effects and reports show that exercise plays an important role in many organizations. In particular, exercise reduces levels of inflammatory products, such as TNF-*α* and IL-6, and reduces the response to cytokines through several mechanisms (15,16). IL-6 is present in a variety of cancers, such as colorectal and breast cancers, and is associated with a poor prognosis in cancer (8).

IL-6 knockout mice and IL-6 overexpression mice showed a decreased numbers of intestinal polyps in knockout mice and increased intestinal polyps in overexpressed mice, in a breast cancer mouse model (1). The expression of IL-6 was reportedly decreased by treadmill running in a breast cancer mouse model. Cytokines that affect other cancer-forming actions include TNF-*α*, which promotes inflammation and affects tumor formation. Postmenopausal breast cancer survivors were subjected to aerobic exercise for 24 weeks, but there was no change in TNF-*α* (4,17). However, a decrease in TNF-*α* expression in plasma and the colon was reported after 6 weeks of regular running in a mouse model of colorectal cancer. A 12-week home-based exercise program also reported a significant decrease in the expression of TNF-*α* in colorectal cancer patients (18,19). IL-18 is an important regulator of the immune response and is known to be an interferon-γ inducing factor that induces cell resistance to infection. IL-18 was found to be increased in breast cancer patients compared to controls, and it is considered an important factor in inducing breast cancer cell migration (20,21).

C-reactive protein (CRP) is an indicator of systemic inflammatory response and there are increased CRP levels in inflammation, injury, and tumors. It is activated by cytokines (IL-6, IL-1, IL-8, and TNF-*α*) secreted by inflammatory cells. In particular, IL-6 is an important mediator of CRP production and is associated with cancer cell growth; CRP correlates to IL-6 concentrations in tumors. CRP is increased in many cancers, including lymphoma, lung cancer, pancreatic cancer, esophageal cancer, gastric cancer, breast cancer, prostate cancer, and kidney cancer (22–27). After menopause, high-intensity physical activity in women results in a 10% lower risk of breast cancer, moderate-intensity physical activity a 13% lower risk, and walking a 6% lower risk (10). Further, this study provides evidence that in the 12-week running program conducted, TNF-*α* mRNA expression was significantly decreased in the breast cancer exercise group compared to the breast cancer group.

These results can be defined as the physical activity-induced reduction of IL-6, IL-18, TNF-*α*, and CRP in breast cancer mice.

## Conclusion

Moderate exercise can reduce the expression of mRNA levels of TNF-*α*, IL-6, IL-18, and CRP in the livers of breast cancer mice. Moderate exercise needs to be performed to reduce liver inflammation in breast cancer patients.

## Ethical considerations

Ethical issues (Including plagiarism, Informed Consent, misconduct, data fabrication and/or falsification, double publication and/or submission, redundancy, etc.) have been completely observed by the authors.
